# The association between metabolic parameters and evening chronotype and social jetlag in non-shift workers: A meta-analysis

**DOI:** 10.3389/fendo.2022.1008820

**Published:** 2022-11-21

**Authors:** Rui Zhang, Xiaoling Cai, Chu Lin, Wenjia Yang, Fang Lv, Jing Wu, Linong Ji

**Affiliations:** Department of Endocrinology and Metabolism, Peking University People’s Hospital, Beijing, China

**Keywords:** chronotype (morningness-eveningness), circadian misalignment, social jetlag (SJL), obesity, glucose metabolism, Lipid

## Abstract

**Aims:**

The aim of the study was to evaluate the association between evening chronotype and social jetlag (SJL) with obesity, blood glucose and lipid levels in non-shift working adults.

**Methods:**

The databases of MEDLINE, EMBASE and Cochrane Reviews were searched for studies analyzing the metabolic parameters among groups of different chronotypes or SJL until Feb 2022. Weighted mean difference (WMD) and 95% confidence intervals (CI) were used to analyze the association between these parameters and chronotypes or SJL.

**Results:**

A total of 27 studies were included in this meta-analysis. Compared with morning chronotype, the participants with evening chronotype had higher body mass index (BMI) (WMD= 0.44 kg/m^2^, 95%CI, 0.30 to 0.57 kg/m^2^, p<0.001), higher fasting blood glucose level (WMD= 5.83mg/dl, 95%CI, 3.27to 8.38 mg/dl, p<0.001), higher total cholesterol level (WMD= 6.63mg/dl, 95%CI, 0.69 to 12.56 mg/dl, p=0.03), and lower high density lipoprotein cholesterol (HDL-C) level (WMD= -1.80mg/dl, 95%CI, -2.30 to -1.31 mg/dl, p<0.001). Compared with the participants with small SJL, the participants with large SJL had larger waist circumference (WMD= 0.80cm, 95%CI, 0.77 to 0.83cm, p<0.001).

**Conclusions:**

Evening chronotype and SJL were associated with obesity and unfavorable metabolic parameters of glucose and lipid metabolism.

**Systematic Review Registration:**

https://www.crd.york.ac.uk/PROSPERO/, identifier CRD42022303401.

## Introduction

The circadian system is controlled by the master clock located in the suprachiasmatic nuclei of the hypothalamus and is entrained by the environmental factors such as the light-dark cycle. However, people live in modern industrialized societies access to artificial light in 24 hours, and they may exhibit different preference in the circadian rhythm of sleep-wake cycle, which is called chronotype (1). Chronotypes can be basically divided into eveningness and morningness, otherwise known as night owls and early birds. Some people with an evening chronotype may cause circadian misalignment. They suffer from chronic sleep deficiency because they sleep late but still have to wake up early due to social demands. As a result, the behavioral rhythm is mismatched with their endogenous circadian rhythm. The inadequate sleep is often paid back on weekends or free days. This phenomenon leads to “social jetlag”, which is measured as the difference of mid-sleep time between work and free days and reflects the degree of circadian misalignment (2).

It was suggested that the evening chronotype and circadian misalignment might be associated with negative health problems including metabolic disorders. In healthy adults, it was reported that in-laboratory induced circadian misalignment resulted in elevated glucose levels, insulin resistance and increased systemic inflammation (3). Epidemiology studies indicated that people with a late chronotype and short sleep duration were prone to have harmful lifestyle such as sedentary behavior and unhealthy nutrition (4, 5). As a demonstrative example of circadian misalignment, shift work was associated with increased risk of obesity and metabolic syndrome (MetS) (6, 7). However, among the studies with non-shift workers, discrepancy existed in the association between chronotypes and obesity. Several large population-based cross-sectional studies did not find an association between late chronotype and adiposity (8–11). A population-based longitudinal study revealed that the evening energy intake was associated with obesity, regardless of chronotype (12). The causal relationship of evening chronotype and obesity, and the mechanism behind their association were not fully elucidated.

In addition, though a longitudinal study indicated a higher incidence of type 2 diabetes (T2DM) in people with evening chronotype (13), the studies of social jetlag (SJL) had inconsistent results. SJL was only associated with increased risk of MetS and diabetes/prediabetes among participants younger than 61 years old in a population-based cohort (14). Another cohort study found SJL was associated with obesity only in the morning chronotypes (15). While some studies demonstrated the association between late chronotype and poor glycemic control in patients with T2DM (16–18), another study did not indicate an association of SJL and HbA1c levels in diabetic patients (19).

In order to better explore the association between evening chronotype and circadian misalignment with obesity, T2DM, and MetS in non-shift workers, we performed this systemic review and meta-analysis of the published studies to compare the anthropometric and metabolic indicators between different chronotypes and SJL groups.

## Material and methods

### Data sources and searches

Comprehensive searches for published studies until Feb 2022 were performed in the electronic database of MEDLINE, EMBASE and Cochrane Reviews. The search strategy included the following terms: chronotype, social jetlag, metabolism, glucose, BMI, obesity (for a complete list, see **Supplementary Table 1**). The registration number for this meta-analysis in PROSPERO was CRD42022303401. The primary aim of this meta-analysis was to clarify the association between circadian misalignment, indicated by evening chronotype or large SJL, and the anthropometric and metabolic variables of adiposity and MetS in general population as well as the patients with metabolic disorders. The PECOS (Population, Exposure, Control, Outcome, Study design) strategy used to define the research question is shown in **Table 1**.

**Table 1:** PECOS criteria for inclusion of studies.

Parameters	Inclusion criteria
Population	Adults in general population or adults with chronic metabolic disorders including T2DM
Exposure	Evening chronotype Large SJL
Control	Morning chronotype Small SJL
Outcome	Parameters of obesity Parameters of glucose metabolism Parameters of lipid metabolism
Study design	No limitation

T2DM, type 2 diabetes; SJL, social jetlag.

### Assessment of chronotypes and circadian misalignment

Chronotypes could be assessed by validated questionnaires including the Horne-Östberg Morningness-Eveningness Questionnaire (MEQ), the reduced MEQ, and the Munich Chrono Type Questionnaire (MCTQ) (1). The simplified assessment of chronotypes used in some studies included scoring from a single self-reported question “Are you a morning person or an evening person?” or the mid-sleep time on free days (MSF). Chronotypes were categorized into 2 to 5 groups in different studies. In our meta-analysis, we only compared the latest chronotype with the earliest chronotype of each study.

Circadian misalignment describes a variety of circumstances including inappropriately timed sleep and wake or misaligned central and peripheral rhythms (20). SJL is a continuous variable to reflect inappropriately timed sleep and wake, which is calculated as the absolute difference between MSF and mid-sleep time on weekdays. People with a SJL ≥1 hour were usually considered as having circadian misalignment (2). Some studies used both 1 and 2 hours as the cutoffs for categorization of different extent of SJL. We recalculated these data and used SJL≥1 hour to indicate circadian mismatch, and compared with SJL <1h group.

### Study selection

Studies were considered eligible for inclusion if they fulfilled the following inclusion criteria: (i) presented primary outcome as evaluating the association between chronotypes or SJL and the anthropometric and metabolic parameters of MetS in randomized controlled studies (RCTs) and observational studies (cross-sectional studies, cohort studies, or case–control studies); (ii) evaluated the metabolic parameters in different chronotypes or SJL groups separately. Studies were excluded using the following criteria: (i) performed in night shift workers; (ii) performed in children and adolescents; (iii) did not classify chronotypes or SJL in groups; (iv) did not present the continuous metabolic indicators in mean ± standard deviation (SD).

For study selection, two investigators independently screened the titles, abstracts, and full articles according to eligibility criteria. Searches were collected, and duplicates were removed. Any potentially relevant citation or any discrepancy was then reviewed in full-text, and decisions about eligibility were independently verified by a third investigator.

### Data extraction and quality assessment

Full-text articles were assessed and data were extracted, including the following information: (i) author; (ii) year of publication; (iii) region; (iv) study design; (v) methods of determination of chronotype; (vi) population; (vii) baseline characteristics. The main variables for evaluation were: (i) numbers of participants with different chronotype or SJL groups; (ii) means and SD for the anthropometric and metabolic variables of two groups; (iii) numbers of MetS, T2DM and the other metabolic disorders in each group. All data from the included publications were extracted into standardized data tables and independently verified by two investigators.

Qualities of cross-sectional and cohort studies were evaluated by the Newcastle-Ottawa Scale (NOS) (21). The evaluation included selection, exposure/outcome and comparability. High quality studies were scored 6–9 stars. RCTs were evaluated using the Cochrane Collaboration tool (22).

### Data synthesis and analysis

Weighted mean differences (WMD) were calculated with 95% confidence interval (CI) for comparisons of anthropometric and metabolic parameters between morning and evening chronotypes, and large and small SJL groups by using the Inverse Variance statistical method. Odds ratios (ORs) and CIs were calculated for the comparison of the prevalence of T2DM and the other metabolic disorders between groups by the Mantel-Haenszel statistical method. Higgins I^2^ statistic was used to quantify the percentage of total variance in the summary estimate due to between-study heterogeneity. Random-effects models was used when an I^2^ value was more than 50% which represented substantial high levels of heterogeneity, while fixed-effects models were used when an I^2^ value was no more than 50%. Sensitivity analysis was performed by including and excluding small sample size, or low-quality studies, or studies with characteristics different from the others. Publication bias was assessed *via* visual inspection of the funnel plot. The subgroup analysis was made based on the definition of chronotype and different characteristics of the population. The statistical analyses were performed with the Review Manager statistical software package (Version 5.3, Nordic Cochrane Center, Copenhagen, Denmark).

## Results

### Characteristics of studies and quality evaluation

**Figure 1:**
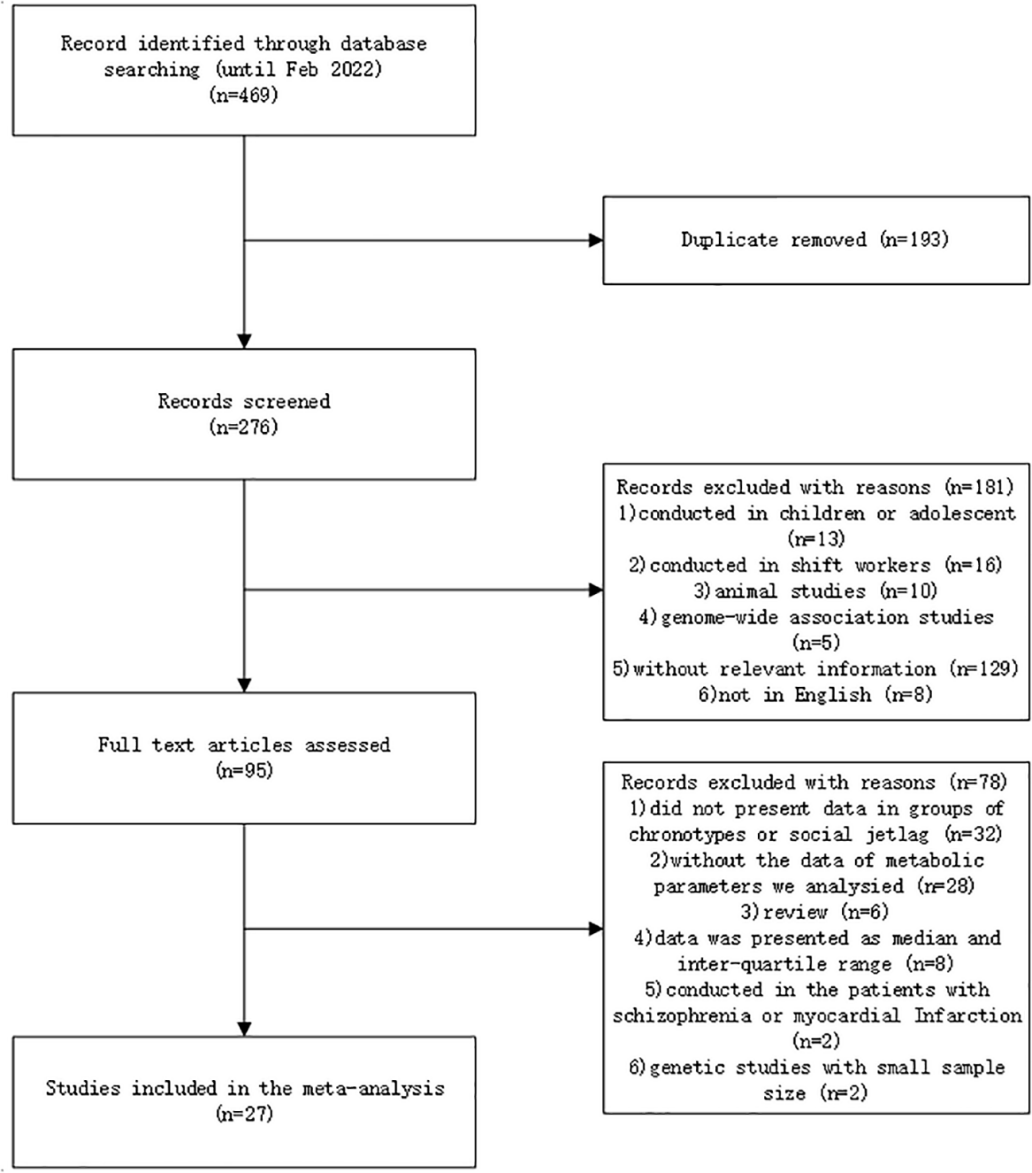
Flowchart of the selection of studies.

A total of 27 studies were included in this meta-analysis, among which 19 were analyzed in groups of chronotypes and 8 were analyzed in the groups of SJL. **Figure 1** showed the flowchart of the included studies. Baseline characteristics of the included studies were presented in **Supplementary Table 2**. RCT was not available in the searching of database. Qualities of the cross-sectional and cohort studies were evaluated by the NOS. Twenty-five studies were evaluated as high quality with a score of 6 to 9 points, and 2 studies were scored as 5 points.

Among the included studies, chronotypes were assessed by validated questionnaires including MEQ (17, 18, 23–30), and the reduced MEQ (12, 13, 31, 32). Some studies used one single question to assess the chronotypes (33, 34). MSF was used in 3 studies as a metric of chronotype (8, 9, 16).

Four among included studies adopted the SJL cutoffs of both 1 and 2 hours to categorize into three groups (14, 15, 19, 35). Three studies used the cutoff of 1 hour to define small and large SJL groups (36–38). One study used the median of SJL, which was 1.07 hours, to define small and large SJL groups (39).

Sixteen studies were conducted in general population or healthy people without chronic diseases, four studies in patients with T2DM, three studies in patients with chronic metabolic disorders, two studies in obese participants, and 2 in subjects who underwent bariatric surgery (**Supplementary Table 2**).

The data of different studies coming from a same cohort were only included once in the meta-analysis of each parameter. There were 4 studies from a large population-based cohort in Finland, two studies coming from a same cohort of Brazil, and two studies from an Italian cohort. Because only two cohort studies of chronotypes and two of SJL provided the data of obesity or metabolic parameters of the follow-up, we could not perform meta-analysis of the follow-up information and only used their baseline data. The prevalence of MetS, hypertension, or the other metabolic disorders, and the mean blood pressure, the fasting insulin levels, insulin resistance indicated by HOMA-IR were not analyzed due to insufficient data.

### The association between chronotypes and adiposity

**Figure 2:**
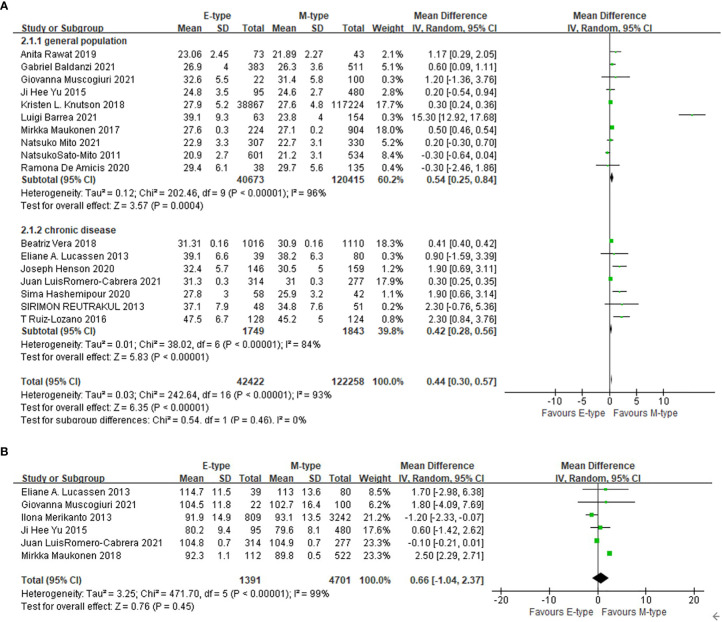
The association between chronotype and adiposity. **(A)** The association between chronotype and BMI in general population and in patients with chronic diseases. **(B)** The association between chronotype and waist circumference.

Compared with the participants with morning chronotype, the mean BMI of the participants with the evening chronotype was higher (WMD= 0.44 kg/m^2^, 95%CI, 0.30 to 0.57 kg/m^2^, p<0.001) (**Figure 2**). In subgroup analysis (**Table 2**), compared with the participants of morning chronotype, the participants of evening type had higher BMI in patients with obesity related chronic disease including T2DM as well as general population (**Figure 2**). The evening chronotype was associated with higher BMI in 12 studies assessed with qualified questionnaire (WMD= 0.59 kg/m^2^, 95%CI, 0.41 to 0.77 kg/m^2^, p<0.001), but not in the five studies with the other assessments of chronotype (WMD= 0.21 kg/m^2^, 95%CI, -0.13 to 0.55 kg/m^2^, p=0.23). Compared with the morning type, the evening type was associated with higher BMI in both subgroups with participants younger or older than the mean age of 50 years old. The mean waist circumference was not significantly different between the evening and morning chronotypes (**Figure 2**).

**Table 2:** Subgroup analysis in the association between chronotype and BMI.

Groups	No. of studies	No. of E-type	No. of M-type	WMD (kg/m^2^)	95%CI (kg/m^2^)	P value	I^2^ (%)
Population							
General population	10	40673	120415	0.54	0.25, 0.84	<0.001	96
With obesity related chronic diseases	7	1749	1843	0.42	0.28, 0.56	<0.001	84
Assessment of chronotype							
Qualified questionnaire	12	2216	3608	0.59	0.41, 0.77	<0.001	95
The other assessment	5	40206	118650	0.21	-0.13, 0.55	0.23	73
Age							
Older than 50 years	11	40572	120295	0.40	0.27, 0.54	<0.001	85
Younger than 50 years	6	1850	1963	2.49	1.23, 3.76	<0.001	97

BMI, body mass index; E-type, evening chronotype; M-type, morning chronotype; WMD, weighted mean difference; CI, confidence interval.

### The association between chronotypes and glycemic indicators

**Figure 3:**
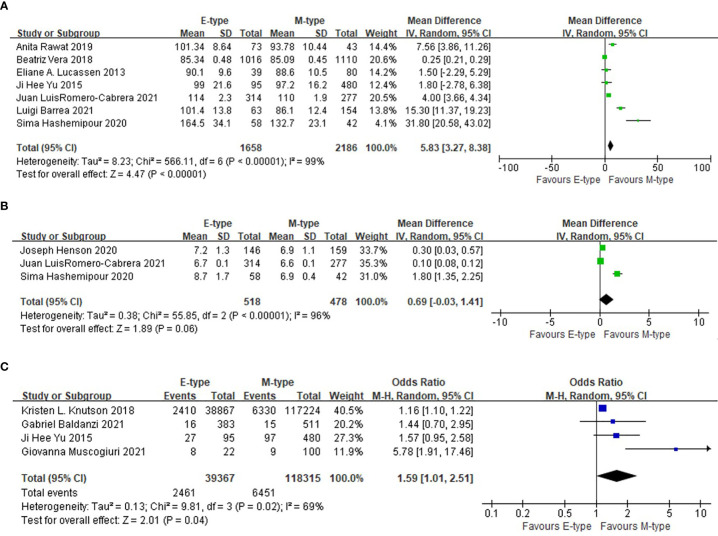
The association between chronotype and glycemic indicators. **(A)** The association between chronotype and fasting blood glucose. **(B)** The association between chronotype and HbA1c. **(C)** The association between chronotype and the prevalence of T2DM.

Compared with the participants with morning type, the participants in the evening type group had higher fasting blood glucose levels (WMD= 5.83mg/dl, 95%CI, 3.27 to 8.38 mg/dl, p<0.001). The association was still significant after excluding one study conducted in T2DM patients (WMD= 4.69mg/dl, 95%CI, 2.14 to 7.24 mg/dl, p<0.001). The prevalence of T2DM was also higher in the evening chronotype group than the morning chronotype (OR= 1.59, 95%CI, 1.01 to 2.51, p=0.04). The mean HbA1c was not statistically different between the two chronotype groups (**Figure 3**).

### The association between chronotypes and lipid levels

**Figure 4:**
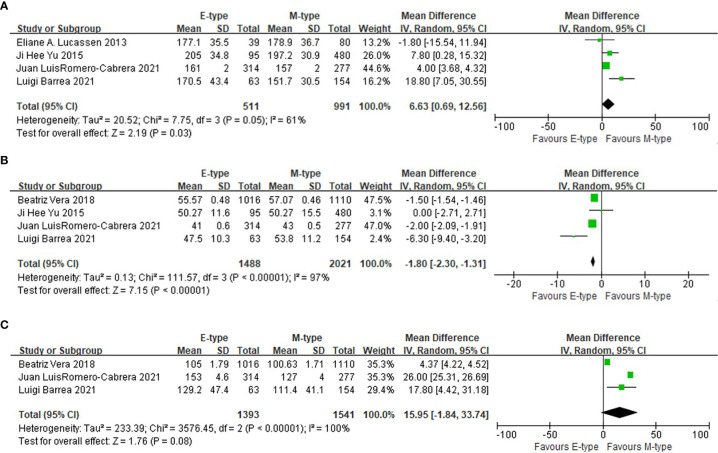
The association between chronotypes and lipids levels. **(A)** The association between chronotypes and total cholesterol. **(B)** The association between chronotypes and HDL-c. **(C)** The association between chronotypes and triglyceride.

Compared with the participants with morning chronotype, the mean total cholesterol level in the evening chronotype group was higher (WMD= 6.63mg/dl, 95%CI, 0.69 to 12.56 mg/dl, p=0.03), and the mean high density lipoprotein cholesterol (HDL-C) was lower (WMD= -1.80mg/dl, 95%CI, -2.30 to -1.31 mg/dl, p<0.001). The triglyceride level was not statistically different between evening and morning chronotypes (**Figure 4**).

### The association between SJL and metabolic parameters

**Figure 5:**
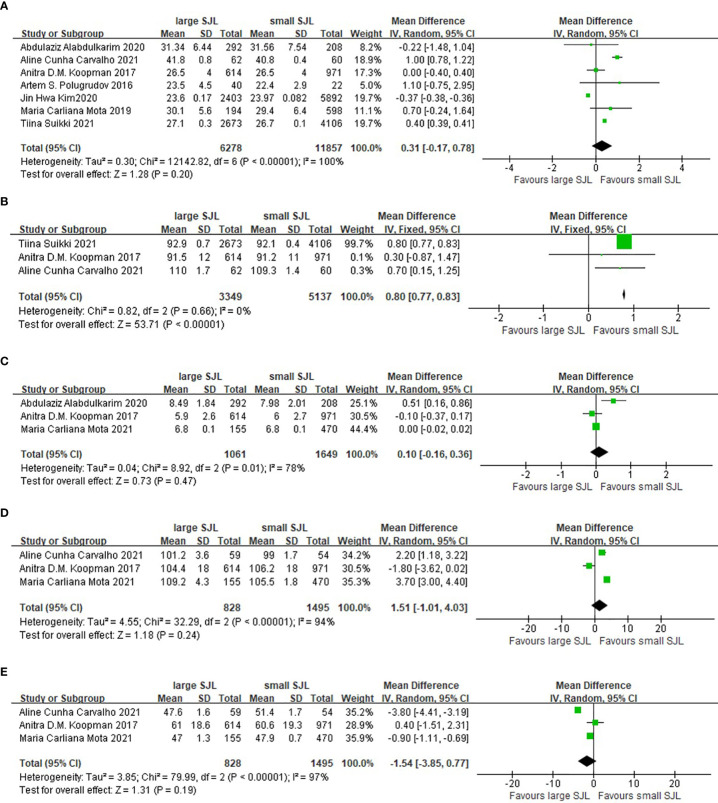
The association between social jetlag and metabolic parameters. **(A)** The association between social jetlag and BMI. **(B)** The association between social jetlag and waist circumference. **(C)** The association between social jetlag and HbA1c. **(D)** The association between social jetlag and fasting blood glucose. **(E)** The association between social jetlag and HDL-c.

Compared with the participants with small SJL, the participants with large SJL had larger waist circumference (WMD= 0.80cm, 95%CI, 0.77 to 0.83cm, p<0.001). However, the mean BMI, fasting blood glucose, HbA1c, and HDL-c levels were not different between small and large SJL groups (**Figure 5**).

## Discussion

According to this meta-analysis, we found that evening chronotype was associated with unfavorable metabolic indicators including higher BMI, higher fasting glucose level, higher total cholesterol level and lower HDL-c level compared with morning chronotype, and larger SJL was associated with larger waist circumference compared with smaller SJL.

Due to the exposure to artificial light and work demand in modern lifestyle, circadian misalignment is a quite common phenomenon. An extreme of circadian misalignment was observed in night shift workers. Studies revealed that shift work increased the risk of abdominal obesity, T2DM, and MetS (6, 7, 40). Among non-shift workers, people with a preference of late chronotype may experience a milder form of circadian misalignment due to the conflict of earlier wake up according to work schedules and later sleep onset in the evening.

In this meta-analysis, we found that evening chronotype was associated with higher BMI compared to morning chronotype in both the general population and participants who already had metabolic disorders including obesity, T2DM and cardiovascular disease (CVD). This result was in line with the observations in some previous cross-sectional studies (17, 31). A longitudinal study further demonstrated that evening chronotype was correlated with lower weight reduction after bariatric surgery (26). In the subgroup analysis, the association between adiposity and evening chronotype was consistent in participants with the age younger and older than 50 years old. This indicated that the association between evening chronotype and adiposity might be independent of age in adults. In line with it, a prospective study among first-year college students also found an association between evening chronotype and weight gain (41).

In addition to obesity, we also analyzed the association between the metabolic parameters of MetS including glucose and lipid levels and evening chronotype. MetS is a cluster of metabolic abnormalities that include hypertension, central obesity, insulin resistance, and dyslipidemia. These metabolic abnormalities share common pathogenies and are strongly associated with an increased risk for developing T2DM and CVD (42). Previous studies demonstrated the association between the prevalence of MetS and late chronotype in general population as well as in patients with CVD (17, 29, 30). Evening chronotype was also associated with a higher prevalence of T2DM in previous cross-sectional studies of middle-aged adults (17, 27). In addition, evening type was found to be associated with higher triglycerides and lower HDL-cholesterol levels in both cross-sectional and longitudinal studies (29, 30). Studies conducted in patients with T2DM and prediabetes demonstrated the association of late chronotype and poor glycemic control (16, 18, 43). A higher HOMA-IR reflecting insulin resistance was also observed in a population-based study (29). In our meta-analysis, the association between evening chronotype and higher fasting glucose level, higher total cholesterol level and lower HDL-c level was in accordance with the previous studies and further supported the relationship between chronotype and MetS

The mechanism of the association between late chronotype and obesity or MetS was partly due to some health-related behaviors. It was revealed that adults with evening chronotype were prone to unhealthy eating habits (5). Data from the UK Biobank project demonstrated that adults with evening chronotype consumed less fruit and vegetables than morning chronotype (44). A study in Finnish population showed greater eveningness was associated with a lower intake of whole grains, whereas a higher intake of alcohol and chocolate (45). People with evening chronotype had a lower adherence to the healthy diets including Baltic Sea diet (10) and Mediterranean diet (31). It was also found that adults with evening chronotype were more likely to skip meals and breakfast in both general population and T2DM patients (46, 47). The other harmful lifestyles including smoking and screen based sedentary behaviors were also related with evening chronotype (4, 44). Similar findings of the association between unhealthy lifestyle and evening chronotype were also found in patients with T2DM and CVD (25, 30). However, the links and/or causal relationships between chronotype and unhealthy eating habits and the other behaviors were not clear. Chronotype may be a causal factor or merely a reflection of a complex set of behaviors that affect eating patterns choice of diet, and sleep quality.

Some people with an evening chronotype may accumulate “sleep debt” during work days, which lead to a greater SJL and circadian misalignment. As a quantifiable indicator of circadian mismatch, SJL was increasingly studied recently. A study conducted in patients with obesity related chronic diseases ascertained the association between larger SJL and higher intake of total calories (37). Larger SJL, which was measured with wrist actigraphy, was associated with higher fasting insulin, waist circumference, and BMI in linear regression models (48). Cross-sectional studies demonstrated that SJL was associated with an increased prevalence of MetS (14, 49). In our meta-analysis, SJL was positively associated with waist circumference, but not with BMI, fasting glucose, HbA1c and HDL-c. The limited number of included studies decreased the significance of this meta-analysis. In addition, the association of SJL and metabolic parameters might be influenced by age and sex. The association of larger SJL and T2DM was more significant in younger (<61 years) participants (14). A longitudinal study found that higher SJL was associated with an increased risk of weight gain only in men (35). Thus, the association between SJL and adiposity or metabolic disorders still needs more investigations in different populations.

The mechanism of the association between circadian misalignment and metabolic disorders is not fully elucidated. In normal physiological state, the circadian rhythms generated by the central clock and phase-adjusted by ambient light should synchronize the clocks in the peripheral tissues to match the daily patterns of behavior, such as feeding, activity and sleep (50). In rodents, peripheral clocks were entrained by timing of food intake (51). In human, the function of peripheral tissues could also be affected by impaired control from the central circadian pacemaker and peripheral clocks. An in-laboratory study conducted in healthy young men found that the delay of meal timing can regulate blood glucose rhythms and clock gene expression in white adipose tissue (52). Thus, in people with circadian mismatch, the misalignment of the peripheral oscillators and the central circadian pacemaker might influence metabolic regulation, and lead to abnormal physiological responses to food intake.

Genetic factors may also play a role in the relationship of circadian misalignment and metabolic dysregulation. Endogenous circadian rhythms generated in circadian clocks are based on a complex program of gene expression (53). Recent accumulating evidence suggested that the clock related genes mutations participated in metabolism including lipogenesis and glucose homeostasis (54, 55). However, a population-based study did not find a relationship between chronotype-related variants and MetS (29). The genetic mechanism of the association between chronotype and metabolic disturbance was still under investigation.

The study had the strength in that it was the first meta-analysis to assess the association between evening chronotype and circadian misalignment and parameters of MetS in non-shift working adults. The limitations of the study were as follows. Firstly, the chronotypes were evaluated by different methods in these included studies causing heterogeneity. However, we have done subgroup analysis to diminish this influence. Secondly, the included studies inconsistently categorized the chronotypes into different number of groups. We only included part of the data in some studies and compared the latest chronotype with the earliest type. Thirdly, the included data were all from cross-sectional observations. We could not evaluate the causal relationship. Fourthly, patients with T2DM and the other chronic diseases were included in the meta-analysis of glycemic indicators and lipid levels. The medication of anti-diabetic or lipid-lowering agents may become confounding factors. However, most included studies were qualified in reducing selection bias between groups. Subgroup analysis of different population was also performed. Lastly, a few relevant studies did not present data in groups classified by chronotypes or SJL, so we could not include them in the meta-analysis.

## Conclusions

In conclusion, this meta-analysis demonstrated that an evening chronotype and circadian misalignment presented as larger SJL, were associated with several unfavorable metabolic parameters relating to adiposity, glucose and lipid metabolism. In the future, more prospective studies and random controlled trials will be needed to establish causality and elucidate the underlying mechanisms.

## Data availability statement

The raw data supporting the conclusions of this article will be made available by the authors, without undue reservation.

## Author contributions

RZ, CL, and WY performed the study selection and data extraction. RZ and FL performed the statistical analyses. JW helped in the preparation of the tables and figures. RZ and XC wrote the manuscript. XC and LJ designed the systematic review protocol and revised the manuscript. All authors read and approved the final manuscript. All authors contributed to the article and approved the submitted version.

## Funding

This work was supported by Beijing Natural Science Foundation (No.7202216), National Natural Science Foundation of China (Nos. 81970698), and National Key Research and Development Program of China (2018YFC1314100).

## Conflict of interest

The authors declare that the research was conducted in the absence of any commercial or financial relationships that could be construed as a potential conflict of interest.

## Publisher’s note

All claims expressed in this article are solely those of the authors and do not necessarily represent those of their affiliated organizations, or those of the publisher, the editors and the reviewers. Any product that may be evaluated in this article, or claim that may be made by its manufacturer, is not guaranteed or endorsed by the publisher.

## Abbreviations

BMI, body mass index; CVD, cardiovascular disease; CI, confidence interval; HbA1c, glycated hemoglobin A1c; HDL-c, high density lipoprotein cholesterol; HOMA-IR, homeostatic model assessment of insulin resistance; MCTQ, Munich Chrono Type Questionnaire; MetS, metabolic syndrome; MEQ, Horne-Östberg Morningness-Eveningness Questionnaire; MSF, mid-sleep time on free days; NOS, Newcastle-Ottawa Scale; OR, Odds ratios; RCT, randomized controlled studies; SD, standard deviation; SJL, social jetlag; T2DM, type 2 diabetes mellitus; WMD, weighted mean difference.
