# Supplementary material for: The association between metabolic parameters and evening chronotype and social jetlag in non-shift workers: A meta-analysis
Source: Front Endocrinol (Lausanne). 2022 Nov 21;13:1008820. doi: 10.3389/fendo.2022.1008820 (PMC9720311; doi:10.3389/fendo.2022.1008820)
Supplement: Supplementary file 1 [file Table_1.docx]

Supplementary table 1. The search strategy terms

| Population | Exposure/control | Outcome | Study design |
| --- | --- | --- | --- |
| NOT “Children” OR “adolescent” OR “shift worker” | “Chronotype” OR “circadian misalignment” OR “morningness” OR “eveningness” OR “nocturnal” OR “diurnal” OR ”circadian mismatch” OR “social jetlag” OR “sleep irregularity” | “metabolism” OR “glucose metabolism” OR ”lipid” OR “glucose” OR “obesity” OR “body mass index” OR “waist circumference” OR “adiposity” OR “diabetes” OR “Type 2 diabetes” OR “insulin resistance” OR “metabolic syndrome” OR “hypertension” OR “blood pressure” | No limitation |
