# Supplementary material for: The association between metabolic parameters and evening chronotype and social jetlag in non-shift workers: A meta-analysis
Source: Front Endocrinol (Lausanne). 2022 Nov 21;13:1008820. doi: 10.3389/fendo.2022.1008820 (PMC9720311; doi:10.3389/fendo.2022.1008820)
Supplement: Supplementary file 2 [file Table_2.docx]

**Title: The association of metabolic parameters and circadian misalignment: a meta-analysis**

Rui Zhang, Xiaoling Cai, Chu lin, Wenjia Yang, Fang Lv, Jing Wu, Linong Ji

**Supplementary table 2. Characteristics and quality evaluation of included studies.**

| Author | Year | Country | Study design | Participants | Chronotype assessment | Categorization | No. | Age | Sex, female | Sleep duration | selection | comparability | exposure | Total points |
| --- | --- | --- | --- | --- | --- | --- | --- | --- | --- | --- | --- | --- | --- | --- |
| SIRIMON REUTRAKUL | 2013 | USA | Cross-sectional | Patients with T2DM | mid-sleep time on free days (MSF) | 4 groups by quartiles of  MSF | 51  46  49  48 | 60.5±11.7  61.2±11.2  56.1±14.7  55.7±13.5 | 38 (74.5)  29(63.0)  31 (63.2)  37 (77.1) | 8.0 (2.0–20.0)  9.5 (4.9–19.2)  15.0 (5.0–22.8)  14.0 (8.0–18.0) | 2 | 1 | 3 | 6 |
| Ji Hee Yu | 2015 | Korea | Cross-sectional | General population | Horne-Ostberg Morningness-Eveningness Questionnaire (MEQ) | morning (59–86), neither (42–58), or evening (16–41) type | 480  1045  95 | 53.7±3.0  52.7±3.0  52.2±2.7 | 218(45.4)  544(52.1)  58(61.1) | 6.8±1.1  6.8± 1.1  6.7± 1.4 | 3 | 1 | 3 | 7 |
| Anita Rawat | 2019 | India | Cross-sectional | General population | MEQ | Definite morning, intermediate, definite evening group | 43  87  73 | 18.05±0.65  18.90±0.68  18.21±0.67 | 16 (37.21)  30 (34.48)  26 (35.62) | ＜6h, n(%)  8 (18.6)  61 (70.1)  50 (68.5) | 2 | 1 | 3 | 6 |
| Sima Hashemipour | 2020 | Iran | Cross-sectional | Patients with T2DM | MEQ | 3 groups of morning,  intermediate or evening | 42  40  58 | 40.5±3.4  40.8±3.8  40.8±3.0 | 23(54.8)  21(52.5)  37(63.8) | 7.9±0.8 7.1±0.9 7.3±1.5 | 2 | 1 | 3 | 6 |
| Eliane A. Lucassen | 2013 | USA | Cross-sectional | Obese and short sleeping participants | MEQ | Morning (score: 50–86) or Evening  types (score: 16–49) | 80  39 | 41.7±5.9  38.6±7.8 | 61(76)  31(80) | Working days (min)  339±52  346±64  Non-working days(min)  386±61  379±60 | 2 | 1 | 3 | 6 |
| Joseph Henson | 2020 | UK | Cross-sectional | Patients with T2DM | MEQ | morning type (scores≥65),  intermediate type (53-64) or evening type (score of ≤52) | 159  330  146 | 64.3±7.7  64.9±7.6  60.5±9.9 | 57 (35.8)  96 (29.0)  67 (45.9) | 383.8 (369.4 to 398.2)  391.4 (379.3 to 403.4)  376.1 (361.8 to 390.5)  (min) | 3 | 1 | 2 | 6 |
| Ramona De Amicis | 2020 | Italy | Cross-sectional | General population | A shortened 5-item version of MEQ | extreme M-Types (scores> 17)  Intermediate N-Types (12–17  )  extreme E-Types (< 12 points) | 135  243  38 | 51±12  48±13  51±13 | 96 (71.1)  163 (67.1)  30 (78.9) | Not report | 4 | 1 | 3 | 8 |
| Mirkka Maukonen^a^ | 2018 | Finland | Cohort  follow-up 7 years | General population | a shortened  six-item version of MEQ | morning  type (19–27 points), intermediate (13–18 points) and  evening type  (6–12 points) | 552  433  112 | Baseline  55.7±0.5  51.0±0.6  47.3±1.2 | Baseline  293(53.1)  254(58.7)  72(64.3) | Baseline  7.2±0.0  7.4±0.0  7.1±0.1 | 4 | 1 | 3 | 8 |
| Mirkka Maukonen^a^ | 2017 | Finland | Cross-sectional | General population | a shortened  six-item version of MEQ | morning type (19-27 points), intermediate (13-18 points), and evening type (6-12 points) | 904  726  224 | 53.4±0.4  48.4±0.5  43.9±0.9 | 453(50.1)  314(43.3)  84(37.5) | Sleep ≥10 h/night (%)  0.9  1.9  4.1  Sleep ≤5 h/night (%)  3.8  3.6  5.5 | 4 | 1 | 3 | 8 |
| T Ruiz-Lozano | 2016 | Spain | Cohort  Follow-up 72 months | subjects who underwent bariatric surgery | MEQ | morning  (＞64 of score), neutral (53–64 of score),  or evening (＜53 of score) types | M-type  124  E-type  128 | Baseline  Total  52.0±11.0 | Baseline  Total  199(79) | Baseline  Total  6.98±1.42 | 3 | 1 | 3 | 7 |
| Kristen L. Knutson | 2018 | UK | Cohort  Follow-up 6.5 years | General population | a single self-reported question | 4 categories:  definite morning types, moderate morning types, moderate evening types, and definite evening types | 117224  153895  123282  38867 | Baseline  57.4±7.8  56.8±8.0  55.8±8.3  55.1±8.3 | Baseline  66232(56.5)  86489(56.2)  68052(55.2)  20716(53.3) | Baseline  7.1±1.1  7.2±1.0  7.2±1.1  7.1±1.2 | 4 | 1 | 3 | 8 |
| Giovanna Muscogiuri^b^ | 2021 | Italy | Cross-sectional | General population  middle-aged subjects | MEQ | morning (59–86), neither (42–58), or evening  (16–41) type | 100  50  22 | 55.5±13.7  43.0±17.4  55.3±11.9 | 66(66.0)  37(74.0)  20(90.9) | 5.5±4.3  7.0±4.9  5.27±2.47 | 3 | 1 | 2 | 6 |
| Luigi Barrea^b^ | 2021 | Italy | Cross-sectional | General population | MEQ | morning (59–86 points), neither (42–58), or  evening (16–41) chronotype | 154  30  63 | 38.5±11.0  40.0±11.5  30.3±8.2 | 113(73.4) 18(60.0) 16(25.4) | Not report | 2 | 1 | 3 | 6 |
| Gabriel Baldanzi | 2021 | Sweden | Cross-sectional | General population | a single self-reported question | 5 categories  extreme morning-type; moderate  morning-type; intermediate; moderate evening-type; and extreme evening-type | 511  391  845  306  383 | 60.8±8.3  60.2±8.262.3±8.4  60.1±8.7  61.6±8.1 | 262(51.3)  192(49.1)  435(51.5)  140(45.8)  200(52.2) | 6.8±1.0  6.9±0.9  6.9±1.0  6.9±0.9  6.9±1.0 | 4 | 1 | 2 | 7 |
| Beatriz Vera | 2018 | Spain | Cross-sectional | overweight and obese subjects | MEQ | Morning (≥53 points), evening (<53) | 1110  1016 | 42.97±12.67  36.17±12.68 | 902 (81.3)  820 (80.7) | 7.53±0.04  7.52±0.04 | 3 | 1 | 3 | 7 |
| NatsukoSato-Mito | 2011 | Japan | Cross-sectional | Students of universities and colleges | mid-sleep time | quintiles from the earliest (Q1) to the latest quintile (Q5) | 534  763  669  737  601 | 18.1±0.3 | All | 6.41±1.22  6.41±1.00  6.31±1.14  6.46±1.34  8.07±1.49 | 3 | 1 | 3 | 7 |
| Natsuko Mito | 2021 | Japan | Cross-sectional | women of the grandmothers’  generation | mid-sleep time | quintiles from the earliest (Q1) to the latest quintile (Q5) | 330  285  361  335  307 | 74.4±4.8  74.8±4.7  74.6±5.0  74.5±4.9  74.6±5.0 | All | 7.47±1.07  7.44±1.13  7.25±1.04  7.26±1.08  7.15±1.15 | 3 | 1 | 3 | 7 |
| Juan LuisRomero-Cabrera | 2021 | Spain | Cross-sectional | patients with established cardiovascular heart disease and high cardiovascular risk | MEQ | morning-type (scoring >61), intermediate (56 -61), evening (<56) | 277  266  314 | 60±0.5 60±0.5 58±0.5 | 37 (13.9)  38 (14.3)  72 (22.9) | 7:42  7:48  7:54 | 3 | 1 | 3 | 7 |
| Ilona Merikanto^a^ | 2013 | Finland | Cross-sectional | General population | a shortened six-item version of MEQ | definitely or moderately E-types (5 to 12 points), I-type (13 to 18), and definitely or moderately M-types (19 to 27) | 3242  2807  809 | 54.4±12.5  50.2±13.7  45.9±13.5 | 1644(50.7)  1617(57.6)  483(59.7) | ≤6h, n(%)  411(18.6)  292(15.7)  108(22.0)  ≥9h, n(%)  152(6.9)  160(8.6)  55(11.2) | 4 | 1 | 3 | 8 |
| Tiina Suikki^a^ | 2021 | Finland | Cross-sectional | General population | a shortened six-item version of MEQ | M-type/SJL＜1  M/SJL1-2  M/SJL≥2  I-type/SJL＜1  I/SJL1-2  I/SJL≥2  E-type/SJL＜1  E/SJL1-2  E/SJL≥2 | 1951  858  157  1682  977  218  473  352  111 | 59.6±0.3  51.6±0.4  48.3±1.0  57.2±0.3  47.8±0.4  44.1c0.9  54.2±0.6  44.2±0.7  39.6±1.3 | 1005(51.50)  474(55.20)  85(54.10)  932(55.40)  558(57.10)  122(55.80)  303(64.10)  218(61.90)  67(60.40) | 7.4±0.02  7.4±0.04  7.5±0.08  7.5±0.03  7.5±0.037.4±0.07  7.5±0.06  7.4±0.07  7.4±0.12 | 3 | 1 | 3 | 7 |
| Aline Cunha Carvalho | 2021 | Brazil | Cohort  Follow-up for 6 months | patients of bariatric surgery | - | small SJL and large SJL classified by the median SJL 1:07 | 60  62 | Baseline  33.5 (28.0–39.0)  33.0 (28.0–39.0) | Baseline  48 (80.0)  46 (74.2) | Not report | 2 | 1 | 3 | 6 |
| Jin Hwa Kim | 2020 | Korea | Cross-sectional | General population | - | 3 groups according to SJL: <1 h, 1–2 h and >2 h | 5892  1960  443 | 50.7±0.42  40.8±0.48  32.4±0.83 | 3425(58.1)  1222(62.3)  231(52.1) | ≥7 h (%) male  66.4 75.5 72.3  Female  63.8 76.9 80.1 | 3 | 1 | 2 | 6 |
| Maria Carliana Mota^c^ | 2021 | Brazil | Cohort  Follow-up for 1 year | Patient with non-communicable  chronic diseases | - | SJL ≤ 1 h or > 1 h | 470  155 | Baseline  58.2±11.2  49.5±12.2 | Baseline  353 (75.1)  123 (79.4) | Baseline  7.2±1.3 7.6±1.6 | 2 | 1 | 3 | 6 |
| Maria Carliana Mota^c^ | 2019 | Brazil | Cross-sectional | Patient with obesity-related chronic diseases | - | SJL ≤ 1h or >1h | 598  194 | 57.9±11.6  49.8±12.7 | 433 (72.5)  148 (76.2) | 07.20±01:40 07:30±02:00 | 2 | 1 | 2 | 5 |
| Anitra D.M. Koopman | 2017 | Netherlands | Cross-sectional | General population | Munich ChronoType Questionnaire (MCTQ) | 3 groups: no SJL (<1  h), 1-2 h SJL, or >2 h SJL | 971  497  117 | 64 ± 6  57 ± 5  57 ± 5 | 524(54)  263(53)  48(41) | 8.2±1  7.8±1  7.8±1 | 3 | 1 | 3 | 7 |
| Abdulaziz Alabdulkarim | 2020 | Saudi Arabia | case-control | Patient with diabetes | MCTQ | SJL＜1  SJL 1-2h  SJL≥2h | 208  111  181 | 50.21±11.06  47.79±10.82  44.29±13.55 | 108 (45)  45 (18.75)  87 (36.25) | Not report | 2 | 1 | 3 | 6 |
| Artem S. Polugrudov | 2016 | Russia | Cross-sectional | practically healthy young persons | MCTQ | SJL ≤0.99 h and SJL ≥1.0 h | 22  40 | 22 ± 2 | 12(54.5)  23(57.5) | 8.0 ± 1.4 7.1 ± 1.2 | 2 | 1 | 2 | 5 |

^a b c^ From the same study cohort, did not include in the same meta-analysis.

Abbreviations: MEQ, Horne-Ostberg Morningness-Eveningness Questionnaire; MCTQ, Munich ChronoType Questionnaire; SJL, social jetllag
